# Evaluation of Treatment Outcomes Using dNLR and GNRI in Combination Therapy With Atezolizumab and Bevacizumab for Hepatocellular Carcinoma

**DOI:** 10.1002/cam4.70618

**Published:** 2025-01-22

**Authors:** Atsushi Naganuma, Satoru Kakizaki, Atsushi Hiraoka, Toshifumi Tada, Takeshi Hatanaka, Kazuya Kariyama, Joji Tani, Masanori Atsukawa, Koichi Takaguchi, Ei Itobayashi, Shinya Fukunishi, Kunihiko Tsuji, Toru Ishikawa, Kazuto Tajiri, Hidenori Toyoda, Chikara Ogawa, Hiroki Nishikawa, Takashi Nishimura, Kazuhito Kawata, Hisashi Kosaka, Masashi Hirooka, Yutaka Yata, Hideko Ohama, Hidekatsu Kuroda, Tomomitsu Matono, Tomoko Aoki, Yuki Kanayama, Kazunari Tanaka, Fujimasa Tada, Kazuhiro Nouso, Asahiro Morishita, Akemi Tsutsui, Takuya Nagano, Norio Itokawa, Tomomi Okubo, Taeang Arai, Michitaka Imai, Shinichiro Nakamura, Hirayuki Enomoto, Masaki Kaibori, Yoichi Hiasa, Masatoshi Kudo, Takashi Kumada

**Affiliations:** ^1^ Department of Gastroenterology NHO Takasaki General Medical Center Takasaki Japan; ^2^ Department of Clinical Research NHO Takasaki General Medical Center Takasaki Japan; ^3^ Department of Gastroenterology and Hepatology Gunma University Graduate School of Medicine Maebashi Japan; ^4^ Gastroenterology Center Ehime Prefectural Central Hospital Matsuyama Japan; ^5^ Department of Internal Medicine Japanese Red Cross Himeji Hospital Himeji Japan; ^6^ Department of Gastroenterology Gunma Saiseikai Maebashi Hospital Maebashi Japan; ^7^ Department of Gastroenterology Okayama City Hospital Okayama Japan; ^8^ Department of Gastroenterology and Neurology Kagawa University Kita‐gun Japan; ^9^ Division of Gastroenterology and Hepatology, Department of Internal Medicine Nippon Medical School Tokyo Japan; ^10^ Department of Hepatology Kagawa Prefectural Central Hospital Takamatsu Japan; ^11^ Department of Gastroenterology Asahi General Hospital Asahi Japan; ^12^ Division of Hepatobiliary and Pancreatic Diseases, Department of Gastroenterology Hyogo Medical University Nishinomiya Japan; ^13^ Center of Gastroenterology Teine Keijinkai Hospital Sapporo Japan; ^14^ Department of Gastroenterology Saiseikai Niigata Hospital Niigata Japan; ^15^ Department of Gastroenterology Toyama University Hospital Toyama Japan; ^16^ Department of Gastroenterology and Hepatology Ogaki Municipal Hospital Ogaki Japan; ^17^ Department of Gastroenterology and Hepatology Takamatsu Red Cross Hospital Takamatsu Japan; ^18^ Department of Gastroenterology Osaka Medical and Pharmaceutical University Osaka Japan; ^19^ Hepatology Division, Department of Internal Medicine II Hamamatsu University School of Medicine Hamamatsu Japan; ^20^ Department of Hepatobiliary Surgery Kansai Medical University Hirakata Japan; ^21^ Department of Gastroenterology and Metabology Ehime University Graduate School of Medicine Matsuyama Japan; ^22^ Department of Gastroenterology Hanwa Memorial Hospital Osaka Japan; ^23^ Department of Gastroenterology Takarazuka City Hospital Takarazuka Japan; ^24^ Division of Gastroenterology and Hepatology, Department of Internal Medicine Iwate Medical University Iwate Japan; ^25^ Department of Gastroenterology Hyogo Prefectural Harima‐Himeji General Medical Center Himeji Japan; ^26^ Department of Gastroenterology and Hepatology Kindai University Faculty of Medicine Osaka Japan; ^27^ Gifu Kyoritsu University Ogaki Japan

**Keywords:** dNLR, GNRI, hepatocellular carcinoma, immune checkpoint inhibitor, prognosis

## Abstract

**Aim:**

This study aims to investigate the clinical utility of the derived neutrophil‐to‐lymphocyte ratio (dNLR) and the Geriatric Nutritional Risk Index (GNRI) in predicting treatment outcomes for patients with unresectable hepatocellular carcinoma (HCC) undergoing combination therapy with atezolizumab and bevacizumab (Atez/Bev).

**Methods:**

A retrospective analysis was conducted on 310 patients. The dNLR, NLR, and GNRI were calculated, and their impact on progression‐free survival (PFS) and overall survival (OS) was assessed. The formula for calculating dNLR is: (neutrophil count ÷ [white blood cell count—neutrophil count]), which means it does not require lymphocyte count. Furthermore, GNRI‐dNLR and GNRI‐NLR scores were defined, and their prognostic values were also analyzed.

**Results:**

The median PFS of this cohort was 7.2 months (95% CI: 5.9–8.5), and the median OS was 24.9 months (95% CI: 19.6–30.2). The dNLR, NLR, and GNRI were significant predictors of both PFS and OS. The dNLR showed a significant correlation with the NLR (Pearson correlation coefficient, *p* < 0.0001). Patients with high GNRI‐dNLR scores demonstrated significantly worse PFS and OS compared to those with low scores (*p* = 0.001, *p* < 0.001, respectively). Compared to stratification by GNRI alone, the GNRI‐dNLR or GNRI‐NLR provided better stratification for both PFS and OS.

**Conclusion:**

The dNLR could be a valuable substitute for NLR as a prognostic marker in patients with unresectable HCC undergoing Atez/Bev therapy. It offers a feasible alternative for databases lacking lymphocyte count information, ensuring comprehensive patient stratification and outcome prediction. The GNRI‐NLR or GNRI‐dNLR score provided better stratification compared to GNRI alone.

## 1 Introduction

Unresectable hepatocellular carcinoma (HCC) remains a challenging malignancy with limited treatment options. The combination treatment with atezolizumab and bevacizumab (Atez/Bev) has emerged as a promising first‐line therapy, showing improved survival outcomes and has become commonly used worldwide [1, 2, 3]. The expression of PD‐L1 [4] and activated Wnt/β‐catenin signaling [5, 6] may be promising biomarkers for predicting the clinical outcomes of immune checkpoint inhibitors [7]. While various biomarkers have been reported and their usefulness recognized [7], there are no established biomarkers to predict the outcomes of this treatment.

Prognostic biomarkers such as the neutrophil‐to‐lymphocyte ratio (NLR) have been established as predictors of survival in unresectable HCC patients treated with Atez/Bev [8]. To calculate the NLR, both the neutrophil and lymphocyte counts are required. In clinical practice, information on lymphocyte counts is sometimes unavailable. As a surrogate for the NLR, the derived NLR (dNLR) has been attracting attention as an index that can be used to analyze databases lacking lymphocyte count information [9]. The formula for calculating dNLR is: (neutrophil count ÷ [white blood cell count—neutrophil count]). In other words, the calculation can be done using neutrophil and white blood cell counts.

Recently, we reported that the ALB‐dNLR score may be useful for predicting the prognosis of patients with unresectable gastric cancer receiving nivolumab therapy [10]. In addition to immune‐related factors such as lymphocytes, it is widely known that nutritional status affects the prognosis of cancer patients. Therefore, it is considered useful to combine immune and nutritional predictors. The Geriatric Nutritional Risk Index (GNRI) reflects nutritional status and sarcopenia, which influence cancer prognosis. We reported that GNRI is a useful biomarker for the treatment of hepatocellular carcinoma [11].

This study aims to evaluate the clinical utility of dNLR in predicting treatment outcomes for patients with unresectable HCC undergoing Atez/Bev therapy. Additionally, we evaluated the utility of combining GNRI with NLRs (GNRI‐dNLR score, GNRI‐NLR score) as predictive indicators for these patients.

## 2 Methods

### 2.1 Participated Patients

**FIGURE 1:**
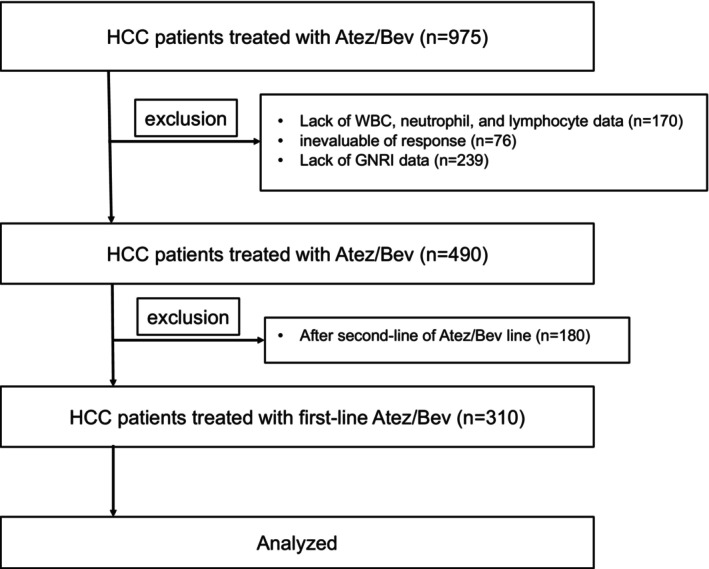
Flowchart of patient selection. Atez/Bev, atezolizumab and bevacizumab; GNRI, Geriatric Nutritional Risk Index.

In this retrospective multicenter study, 975 HCC patients were treated with Atez/Bev in our affiliated hospitals from September 2020 to January 2023. We included first‐line treatment patients and excluded those lacking data on white blood cell counts, neutrophils, lymphocytes, or GNRI. Consequently, a total of 310 patients were included in the present study. The patient selection process is described in Figure 1.

HCC was confirmed through histological diagnosis based on pathological specimens and/or typical findings on radiological imaging, including CT and magnetic resonance imaging. We retrospectively reviewed the medical records and collected patient characteristics, including age, gender, body mass index, etiology of liver disease, performance status (PS), history of systemic therapies, laboratory data, and radiological imaging. Preserved liver function was evaluated using the Child–Pugh classification and the modified albumin–bilirubin (mALBI) grade [12]. Tumor staging was determined based on the Barcelona Clinic Liver Cancer (BCLC) staging system, which considers tumor burden, liver function, and PS [3].

The dNLR was calculated as follows: dNLR = (neutrophil count)/(white blood cell count—neutrophil count) [9]. The GNRI was calculated using the formula: GNRI = (14.89 × serum albumin [g/dL]) + (41.7 × [current weight/ideal weight]) [13]. The cutoff values for NLR were set at 3.0, based on previous reports for HCC [8, 14]. Since the cutoff values of dNLR for HCC have not been established, we set it to be the same as that for NLR. The GNRI‐dNLR score was derived by combining GNRI and dNLR scores. The NLR or dNLR scores were assigned 0 and 1 points for groups lower and higher than the cutoff value, respectively, and the GNRI was assigned a score of 0 for a normal group of 98 or higher and a score of 1 for a group < 98. The GNRI‐dNLR score was calculated by summing both scores, assigning 0 and 1 points to the low group, and 2 points to the high group. The GNRI‐NLR score was also calculated in the same way.

### 2.2 Evaluation on Therapeutic Outcome of Atez/Bev Treatment

The administration of Atez/Bev followed the protocol outlined in the Imbrave150 trial [1], where patients received 1200 mg of atezolizumab intravenously, followed by 15 mg/kg of bevacizumab every 3 weeks. The Atez/Bev treatment was continued until disease progression or unacceptable adverse events occurred. The tumor response was assessed according to the Response Evaluation Criteria in Solid Tumors version 1.1 (RECIST ver.1.1). The best radiological response was classified as complete response (CR), partial response (PR), stable disease (SD), and progressive disease (PD) based on local review. PFS was computed from the date Atez/Bev treatment was initiated to the date of progressive disease or death from any cause, whichever came first. The OS was calculated from the date Atez/Bev treatment was initiated to the date of death from any cause. Adverse events were graded based on The Common Terminology Criteria for Adverse Events version 5.0. Interruption or discontinuation of each drug was carried out according to the guidelines for Atez/Bev treatment provided by the manufacturer.

### 2.3 Statistical Analyses

The categorical variables were described as the number (percentage) and compared using the chi‐squared or Fisher's exact test, as appropriate. The continuous variables were described as the median (interquartile range) and compared using the Mann–Whitney *U*‐test. The PFS and survival curves were drawn using the Kaplan–Meier method and analyzed by a log‐rank test. The following factors were included in the multivariate analyses as explanatory variables such as age, gender, etiology of liver disease, PS, mALBI grade, macroscopic vascular invasion (MVI), extrahepatic spread (EHS), α‐fetoprotein (AFP), des‐gamma‐carboxy prothrombin (DCP), best of response (BOR), and GNRI‐dNLR score/GNRI‐NLR score. All of the reported *p*‐values were two‐sided and *p* < 0.05 were considered statistically significant. All statistical analyses were conducted using EZR Ver. 1.55 (Saitama Medical Center, Jichi Medical University, Saitama, Japan) [15].

## 3 Results

### 3.1 Patient Characteristics

Table 1 shows the patient characteristics of the entire cohort. The patient cohort consisted of 238 males (76.8%) and 72 females (23.2%), with a median age of 74.0 years. A total of 76.5% of the patients had a performance status (PS) of 0. The median body mass index was 23.4 (21.1, 26.3) kg/m^2^. The etiologies of liver diseases were as follows: HBV, *n* = 36 (11.6%); HCV, *n* = 111 (35.8%); alcohol, *n* = 66 (21.3%); and others, *n* = 97 (31.3%). Virus‐related liver diseases accounted for 47.4% of the cases. The Child–Pugh scores were 5 points in 167 patients (53.9%), 6 points in 79 patients (25.5%), and ≥ 7 points in 44 patients (14.2%). The median ALBI score was −2.44 (−2.77, −2.06). Accordingly, 120 (38.7%), 74 (23.9%), 110 (35.5%), and 6 patients (1.9%) were classified as grade 1, 2a, 2b, and 3, respectively. The BCLC stages were determined as follows: very early, *n* = 4 (1.3%); early, *n* = 20 (6.5%); intermediate, *n* = 109 (35.2%); advanced, *n* = 164 (52.9%); and terminal stage, *n* = 12 (3.9%).

**TABLE 1:** Patient characteristics.

Factors	Overall (*n* = 310)
Age	74.0 (68.0, 80.0)
Males, *n* (%)	238 (76.8)
Body mass index (kg/m^2^)	23.4 (21.1, 26.3)
Performance status, *n* (%)	
0	237 (76.5)
1	57 (18.4)
≥ 2	16 (5.1)
Etiology of liver disease, *n* (%)	
HBV	36 (11.6)
HCV	111 (35.8)
Alcohol	66 (21.3)
Others	97 (31.3)
BCLC stage, *n* (%)	
Very early	4 (1.3)
Early	20 (6.5)
Intermediate	109 (35.2)
Advanced	164 (52.9)
Terminal	12 (3.9)
Child–Pugh class, *n* (%)	
A	266 (85.9)
B	43 (13.7)
C	1 (0.3)
mALBI grade, *n* (%)	
1	120 (38.7)
2a	74 (23.9)
2b	110 (35.5)
3	6 (1.9)
ALBI score	−2.44 [−2.77, −2.06]
NLR	2.29 [1.72, 3.46]
dNLR	1.60 [1.23, 2.16]
GNRI	100.2 [92.3, 107.5]
AFP ≥ 100 ng/mL, *n* (%)	103 (33.2)
DCP ≥ 100 mAU/mL, *n* (%)	171 (55.2)

Abbreviations: AFP, α‐fetoprotein; BCLC, Barcelona Clinic Liver Cancer; DCP, des‐γ‐carboxy prothrombin; HBV, hepatitis B virus; HCV, hepatitis C virus; mALBI grade, modified albumin–bilirubin grade; NLR, neutrophil to lymphocyte ratio; dNLR, derived neutrophil to lymphocyte ratio.

### 3.2 Therapeutic Outcome of Atez/Bev Treatment

**FIGURE 2:**
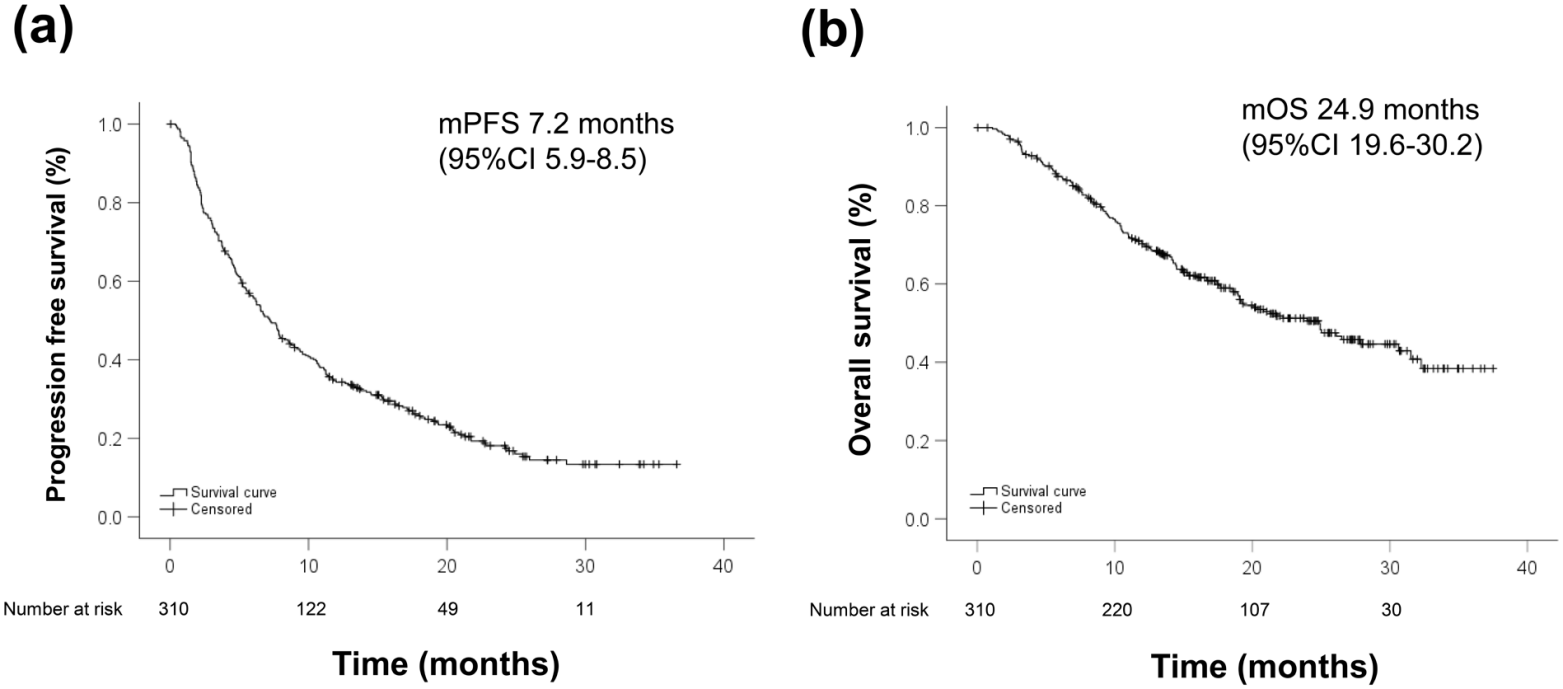
The Kaplan–Meier curves of PFS and OS for all patients. (a) PFS and (b) OS. The median PFS was 7.2 months (95% CI: 5.9–8.5), and the median OS was 24.9 months (95% CI: 19.6–30.2), respectively.

The best radiological response was classified as follows: CR, *n* = 14 (4.5%); PR, *n* = 72 (23.2%); SD, SD, *n* = 150 (48.4%); and PD, *n* = 74 (23.9%). The objective response rate (ORR) was 27.7%, and the disease control rate (DCR) was 76.1%. The Kaplan–Meier curves showed that the median PFS was 7.2 months (95% CI: 5.9–8.5), and the median OS was 24.9 months (95% CI: 19.6–30.2) (Figure 2).

**FIGURE 3:**
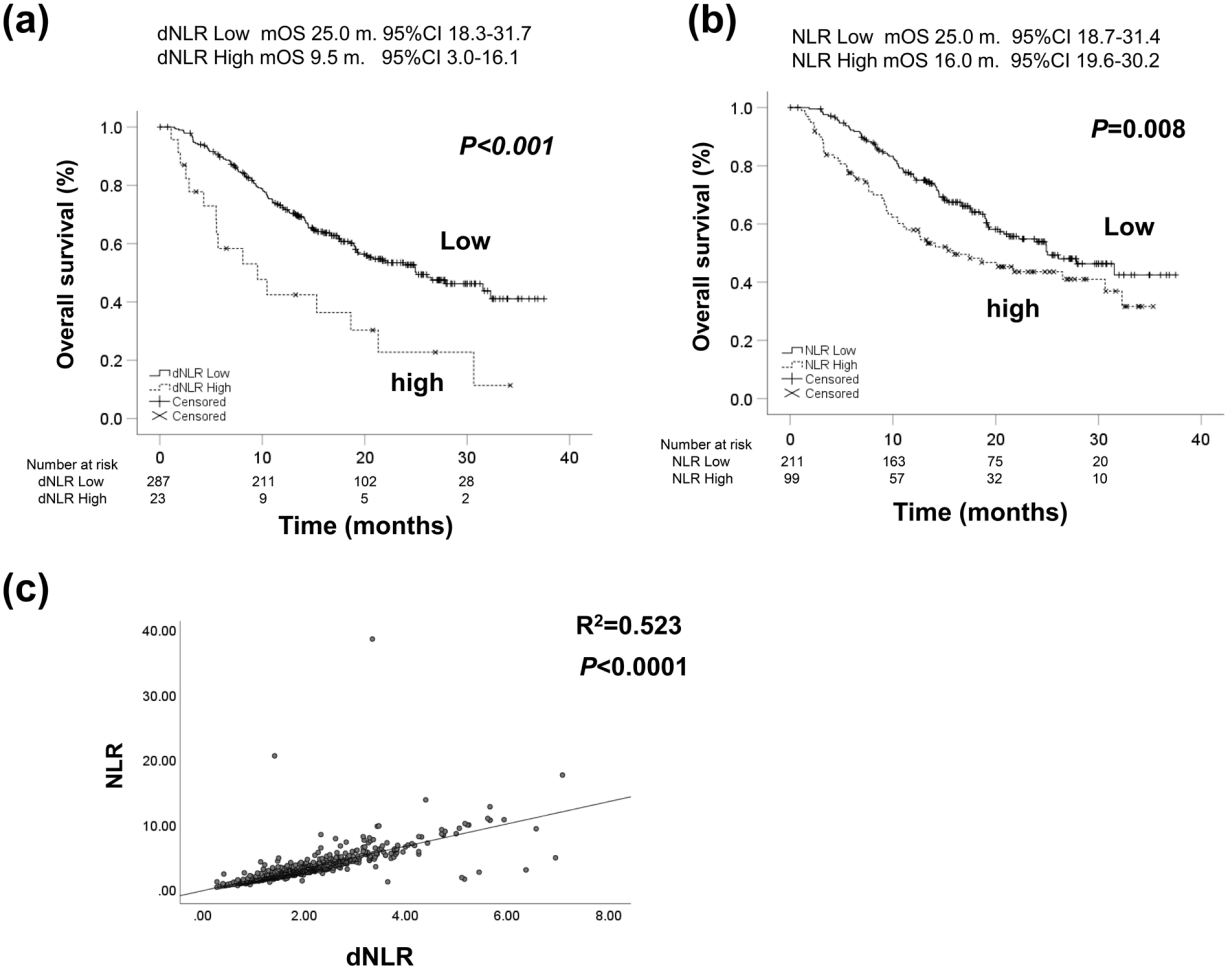
The Kaplan–Meier curves of OS according to dNLR (a) and NLR (b). The median OS of dNLR low group was 25.0 months (95% CI: 18.3–31.7), and that of dNLR high group was 9.5 months (95% CI: 3.0–16.1), respectively (*p* < 0.001). The median OS of NLR low group was 25.0 months (95% CI: 18.7–31.4), and that of NLR high group was 16.0 months (95% CI: 19.6–30.2), respectively (*p* = 0.008). (c) The correlation between dNLR and NLR. Pearson's correlation coefficient was 0.523 and dNLR was significantly correlated with NLR (*p* < 0.0001). dNLR; derived neutrophil‐to‐lymphocyte ratio, NLR; neutrophil‐to‐lymphocyte ratio.

Figure 3 showed the Kaplan–Meier OS curves according to dNLR (a) and NLR (b). The median OS of the low dNLR group was 25.0 months (95% CI: 18.3–31.7), and that of the high dNLR group was 9.5 months (95% CI: 3.0–16.1). OS in the low dNLR group was significantly better than that in the high dNLR group (*p* < 0.001). The median OS of the low NLR group was 25.0 months (95% CI: 18.7–31.4), and that of the high NLR group was 16.0 months (95% CI: 19.6–30.2). OS in the low NLR group was significantly better than that in the high NLR group (*p* = 0.008). Figure 3c showed the correlation between dNLR and NLR. Pearson's correlation coefficient was 0.523 and dNLR was significantly correlated with NLR (*p* < 0.0001).

**FIGURE 4:**
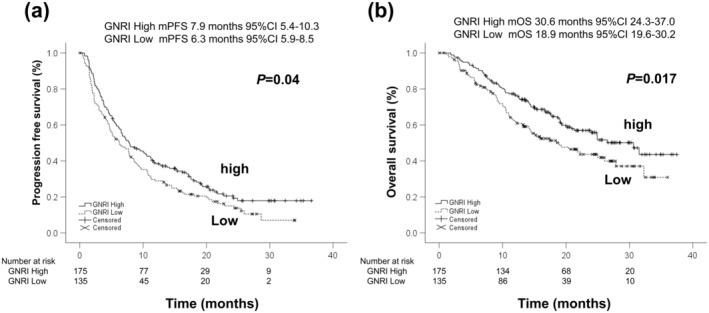
The Kaplan–Meier curves of PFS and OS according to GNRI. (a) PFS and (b) OS. The median PFS of GNRI high group was 7.9 months (95% CI: 5.4–10.3), and that of GNRI low group was 6.3 months (95% CI: 5.9–8.5), respectively (*p* = 0.04). The median OS of GNRI high group was 30.6 months (95% CI: 24.3–37.0), and that of GNRI low group was 18.9 months (95% CI: 19.6–30.2), respectively (*p* = 0.017). GNRI; Geriatric Nutritional Risk Index.

Figure 4 showed the Kaplan–Meier curves of PFS (a) and OS (b) according to GNRI. The median PFS of the high GNRI group was 7.9 months (95% CI: 5.4–10.3), and that of the low GNRI group was 6.3 months (95% CI: 5.9–8.5). PFS of the high GNRI group was significantly better than that of the low GNRI group (*p* = 0.04). The median OS of the high GNRI group was 30.6 months (95% CI: 24.3–37.0), and that of the low GNRI group was 18.9 months (95% CI: 19.6–30.2). OS of the high GNRI group was significantly better than that of the low GNRI group (*p* = 0.017).

**FIGURE 5:**
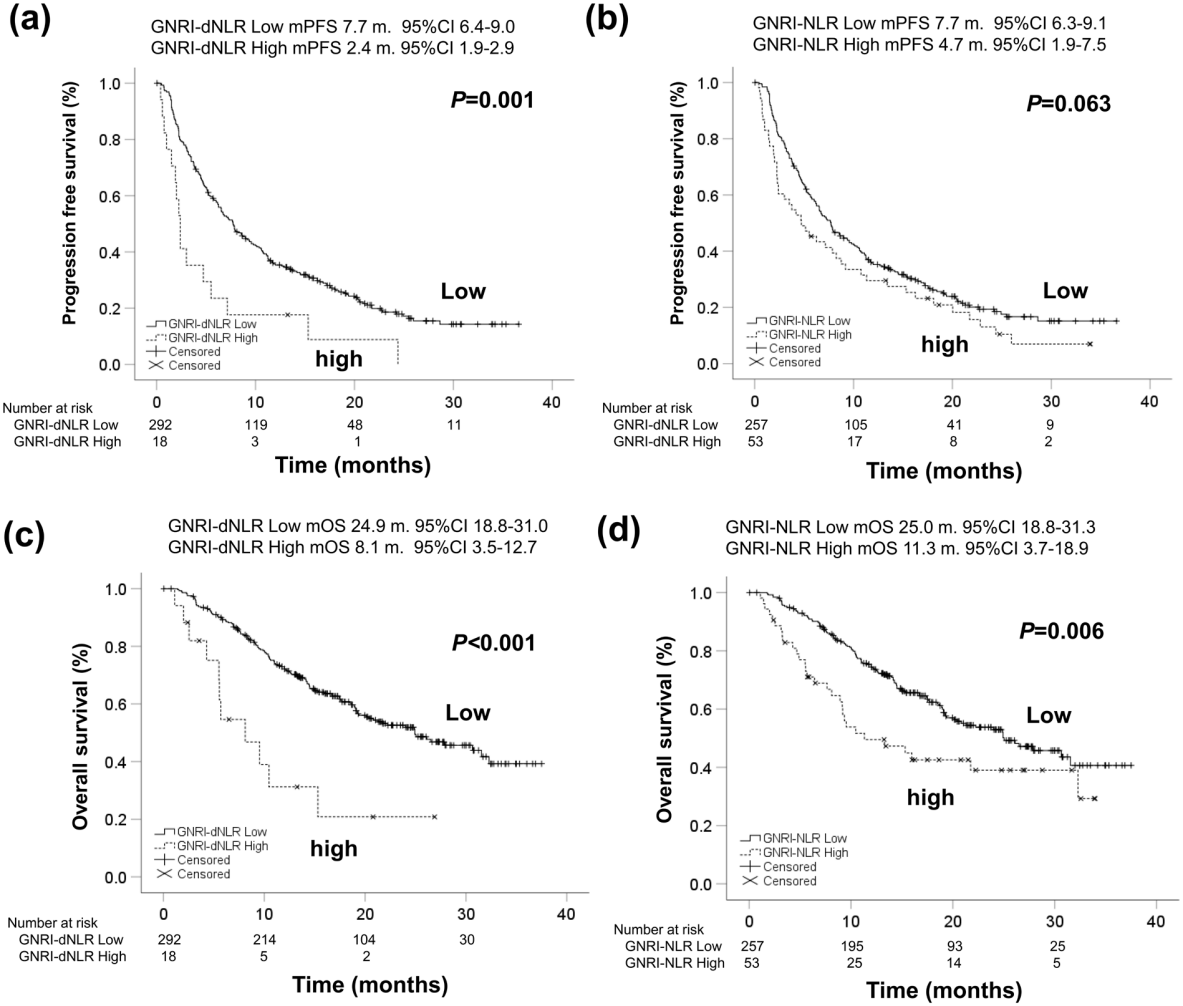
The Kaplan–Meier curves of PFS and OS according to GNRI‐dNLR score and GNRI‐NLR score. (a) PFS according to GNRI‐dNLR score, (b) PFS according to GNRI‐NLR score, (c) OS according to GNRI‐dNLR score, and (d) OS according to GNRI‐NLR score. Median PFS according to GNRI‐dNLR were 7.7 months (95% CI: 6.4–9.0) with low score and 2.4 months (95% CI: 1.9–2.9) with high score groups, respectively (*p* = 0.001). Median PFS according to GNRI‐NLR were 7.7 months (95% CI: 6.3–9.1) with low score and 4.7 months (95% CI: 1.9–7.5) with high score groups, respectively (*p* = 0.063). Median OS according to GNRI‐dNLR were 24.9 months (95% CI: 18.1–31.0) with low score and 8.1 months (95% CI: 3.5–12.7) with high score groups, respectively (*p* < 0.001). Median OS according to GNRI‐NLR were 25.0 months (95% CI: 18.8–31.3) with low score and 11.3 months (95% CI: 3.7–18.9) with high score groups, respectively (*p* = 0.006).

Figure 5 showed the Kaplan–Meier curves of PFS (a, b) and OS (c, d) according to GNRI‐dNLR score and GNRI‐NLR score. Median PFS according to GNRI‐dNLR was 7.7 months (95% CI: 6.4–9.0) in the low score group and 2.4 months (95% CI: 1.9–2.9) in the high score group (*p* = 0.001). Median PFS according to GNRI‐NLR was 7.7 months (95% CI: 6.3–91) in the low score group and 4.7 months (95% CI: 1.9–7.5) in the high score group (*p* = 0.063). There were significant differences in GNRI‐dNLR stratification, although GNRI‐NLR stratification did not show statistically significant differences in PFS.

Median OS according to GNRI‐dNLR was 24.9 months (95% CI: 18.3–31.0) in the low score group and 8.1 months (95% CI: 3.5–12.7) in the high score group (*p* < 0.001). Median OS according to GNRI‐NLR was 25.0 months (95% CI: 18.8–31.3) in the low score group and 11.3 months (95% CI: 3.7–18.9) in the high score group (*p* = 0.006). Both GNRI‐dNLR and GNRI‐NLR scores provided statistically significant stratification for OS.

### 3.3 Factors Associated With PFS and OS

The results of univariate and multivariate analyses associated with PFS are presented in Table 2. Model 1 of the multivariate analyses included the GNRI‐NLR score, and Model 2 included the GNRI‐dNLR score as explanatory variables. The multivariate analyses showed that nonviral etiology, AFP 400 ng/mL or higher, and BOR with CR or PR were significant predictive factors associated with PFS. Both a high GNRI‐NLR score (OR: 1.48, 95% CI: 1.04–2.09, *p* = 0.029) and a high GNRI‐dNLR score (OR: 2.05, 95% CI: 1.15–3.67, *p* = 0.015) were also significant predictive factors associated with poorer PFS.

**TABLE 2:** Univariate and multivariate analyses associated with PFS in patients with hepatocellular carcinoma treated with atezolizumab plus bevacizumab.

Items	Binary variables	Univariate analyses		Multivariate analyses (Model 1)		Multivariate analyses (Model 2)	
		Hazard ratio (95% CI)	*p*	Hazard ratio (95% CI)	*p*	Hazard ratio (95% CI)	*p*
Age	Over 75 years old	1.02 (0.80–1.32)	0.85	0.97 (0.74–1.27)	0.84	0.98 (0.75–1.28)	0.86
Gender	Women	1.11 (0.83–1.49)	0.49	1.03 (0.76–1.39)	0.86	1.05 (0.78–1.42)	0.77
Etiology	Nonviral	1.26 (0.98–1.62)	0.07	1.45 (1.11–1.90)	0.007	1.38 (1.06–1.81)	0.02
PS	One or more	1.53 (1.14–2.04)	0.004	1.16 (0.85–1.60)	0.35	1.15 (0.83–1.58)	0.4
Child–Pugh class	B or C	1.71 (1.20–2.43)	0.003				
mALBI grade	2b or 3	1.50 (1.16–1.95)	0.002	1.32 (1.00–1.73)	0.051	1.30 (0.98–1.71)	0.07
MVI	Vp3/4	2.44 (1.64–3.63)	< 0.001	1.48 (0.95–2.33)	0.09	1.39 (0.88–2.20)	0.15
EHM	Present	1.02 (0.77–1.34)	0.91	0.97 (0.72–1.30)	0.84	0.94 (0.70–1.27)	0.69
AFP	400 ng/mL or more	1.69 (1.26–2.27)	0.001	1.76 (1.25–2.47)	0.001	1.84 (1.31–2.60)	< 0.001
DCP	100 mAU/mL or more	1.55 (1.20–2.01)	0.001	1.18 (0.89–1.56)	0.25	1.20 (0.91–1.58)	0.2
BOR	CR or PR	0.37 (0.27–0.51)	< 0.001	0.32 (0.23–0.45)	< 0.001	0.33 (0.24–0.46)	< 0.001
NLR	Three or more	1.26 (0.96–1.64)	0.09				
dNLR	Three or more	1.84 (1.17–2.87)	0.008				
GNRI	< 98	1.30 (1.01–1.67)	0.041				
GNRI‐NLR score	High	1.35 (0.98–1.87)	0.065	1.48 (1.04–2.09)	0.029		
GNRI‐dNLR score	High	2.29 (1.37–3.80)	0.001			2.05 (1.15–3.67)	0.015

Abbreviations: AFP, α‐fetoprotein; BOR, best of response; CI, confidence interval; CR, complete response; DCP, des‐gamma‐carboxy prothrombin; dNLR, derived neutrophil‐to‐lymphocyte ratio; EHM, extrahepatic metastasis; GNRI, Geriatric Nutritional Risk Index; mALBI grade, modified albumin–bilirubin grade; MVI, major vascular invasion; NLR, neutrophil‐to‐lymphocyte ratio; PR, partial response; PS, performance status.

The results of univariate and multivariate analyses associated with OS are presented in Table 3. The multivariate analyses showed that mALBI grade 2b or 3, AFP 400 ng/mL or higher, and BOR with CR or PR were significant predictive factors associated with OS. Both a high GNRI‐NLR score (OR: 1.60, 95% CI: 1.04–2.47, *p* = 0.03) and a high GNRI‐dNLR score (OR: 2.31, 95% CI: 1.16–4.61, *p* = 0.017) were also significant predictive factors associated with poorer OS.

**TABLE 3:** Univariate and multivariate analyses associated with OS in patients with hepatocellular carcinoma treated with atezolizumab plus bevacizumab.

Items	Binary variables	Univariate analyses		Multivariate analyses (Model 1)		Multivariate analyses (Model 2)	
		Hazard ratio (95% CI)	*p*	Hazard ratio (95% CI)	*p*	Hazard ratio (95% CI)	*p*
Age	Over 75 years old	1.22 (0.87–1.69)	0.25	1.23 (0.86–1.76)	0.26	1.23 (0.86–1.76)	0.26
Gender	Women	1.06 (0.72–1.55)	0.79	1.02 (0.69–1.50)	0.94	1.03 (0.69–1.53)	0.88
Etiology	Nonviral	1.17 (0.84–1.63)	0.35	1.18 (0.84–1.66)	0.35	1.12 (0.80–1.59)	0.51
PS	One or more	1.56 (1.07–2.30)	0.02	1.09 (0.72–1.67)	0.68	1.06 (0.69–1.62)	0.8
Child‐Pugh class	B or C	2.81 (1.84–4.29)	< 0.001				
mALBI grade	2b or 3	2.19 (1.57–3.06)	< 0.001	2.17 (1.51–3.11)	< 0.001	2.13 (1.48–3.07)	< 0.001
MVI	Vp3/4	2.47 (1.54–3.97)	< 0.001	1.30 (0.76–2.25)	0.34	1.28 (0.74–2.20)	0.38
EHM	Present	1.33 (0.94–1.87)	0.11	1.39 (0.97–1.99)	0.08	1.35 (0.94–1.95)	0.11
AFP	400 ng/mL or more	1.89 (1.32–2.71)	0.001	1.81 (1.19–2.74)	0.01	1.92 (1.27–2.92)	0.002
DCP	100 mAU/mL or more	1.49 (1.07–2.09)	0.02	1.06 (0.73–1.55)	0.76	1.08 (0.74–1.57)	0.69
BOR	CR or PR	0.31 (0.19–0.49)	< 0.001	0.24 (0.15–0.40)	< 0.001	0.25 (0.16–0.41)	< 0.001
NLR	Three or more	1.58 (1.13–2.22)	0.01				
dNLR	Three or more	2.55 (1.51–4.29)	< 0.001				
GNRI	< 98	1.49 (1.07–2.07)	0.02				
GNRI‐NLR score	High	1.75 (1.17–2.61)	0.007	1.60 (1.04–2.47)	0.03		
GNRI‐dNLR score	High	3.15 (1.69–5.85)	< 0.001			2.31 (1.16–4.61)	0.017

Abbreviations: AFP, α‐fetoprotein; BOR, best of response; CI, confidence interval; CR, complete response; DCP, des‐gamma‐carboxy prothrombin; dNLR, derived neutrophil‐to‐lymphocyte ratio; EHM, extrahepatic metastasis; GNRI, Geriatric Nutritional Risk Index; mALBI grade, modified albumin‐bilirubin grade; MVI, major vascular invasion; NLR, neutrophil‐to‐lymphocyte ratio; PR, partial response; PS, performance status.

## 4 Discussion

The major findings of the present study indicate that dNLR is a prognostic predictor for Atez/Bev treatment comparable to NLR. Compared with stratification by GNRI alone, the GNRI‐dNLR showed better stratification for PFS and OS. The multivariate analyses showed that high GNRI‐NLR and GNRI‐dNLR scores were significant predictive factors associated with poorer PFS and OS.

Immune checkpoint inhibitors (ICIs) have revolutionized cancer treatment by enhancing the immune response against tumors. Recent studies have explored the relationship between the NLR and the efficacy of ICIs [16]. NLR is an easily measurable biomarker reflecting the balance between pro‐tumor inflammation (neutrophils) and antitumor immunity (lymphocytes). A high NLR is often associated with poor prognosis across various cancers [17]. The proposed mechanism suggests that a lower NLR reflects a more favorable immune environment, potentially enhancing the effectiveness of ICIs in targeting and eliminating cancer cells [17]. A lower baseline NLR was significantly associated with better OS and PFS in HCC patients receiving Atez/Bev [8, 18].

In recent years, the dNLR has been proposed as an alternative marker of NLR [19]. The dNLR can be calculated using only white blood cell and neutrophil counts and does not require information on lymphocyte counts, so it has the advantage of being usable in databases without lymphocyte information. Indeed, in this real‐world cohort, 170 out of 975 cases (17.4%) had missing leukocyte and/or lymphocyte data, making it impossible to calculate NLR. NLR and dNLR were strongly correlated and served as reliable prognostic indicators [20]. However, it is important to note that while dNLR and NLR are closely related, they are not always interchangeable. Some studies have reported that NLR might be slightly more sensitive in detecting subtle changes in the immune status of patients [21]. Notably, our HCC cohort demonstrated that dNLR can serve as a substitute for NLR.

GNRI is calculated using serum albumin levels and body weight and serves as an indicator of nutritional status and frailty, both of which are critical in cancer outcomes. Malnutrition and frailty are often associated with a poor prognosis in HCC due to their impact on immune function and the body's ability to tolerate and respond to treatments [13]. GNRI has been associated with prognosis after hepatic resection [22, 23] and transarterial chemoembolization [24] in HCC patients. We previously reported that GNRI is an effective nutritional prognostic tool for predicting prognosis and muscle volume loss in HCC patients treated with Atez/Bev [11].

We showed that combining GNRI and NLR/dNLR can enhance the prognostic accuracy in this study. A low GNRI and a high NLR had significantly poorer OS compared to patients with more favorable values for either or both markers. This combined approach leverages the predictive strengths of both biomarkers, providing a more comprehensive picture of the patient's immune‐nutritional status. Although not in cancer patients, there are reports that the combination of GNRI and NLR can predict the prognosis of dialysis patients [25]. Malnutrition can weaken the immune system, reducing the effectiveness of ICIs, while a high NLR may indicate a pro‐tumor inflammatory environment that further undermines therapeutic efficacy. Therefore, assessing both GNRI and NLR/dNLR together could help clinicians identify high‐risk patients. Further research is needed to validate the combined use of GNRI and NLR/dNLR across different cancer types and treatment modalities. However, this approach holds promise for optimizing personalized treatment plans, particularly in HCC.

In this study, we used the Child–Pugh classification and mALBI grade prior to Atez/Bev treatment as indicators of liver function. Hepatic reserve is a crucial factor in predicting OS and PFS. However, even if the Child–Pugh classification and mALBI grade values before Atez/Bev treatment are identical, hepatic reserve may differ depending on the patient's prior treatment history (e.g., surgical resection, percutaneous ablation, or transarterial treatments). Therefore, it is essential to develop an appropriate treatment plan that considers not only liver function and tumor factors but also the patient's treatment history [26]. Prognosis prediction requires a comprehensive evaluation that includes liver function values alongside treatment history. Although this study included a group of patients with poor liver function, maintaining liver reserve during systemic drug therapy is critical for ensuring treatment continuity. To achieve this, a multidisciplinary approach is necessary to evaluate both tumor characteristics and liver function comprehensively. Selecting a treatment strategy that optimizes the patient's OS and PFS requires collaborative decision‐making to balance the benefits of therapy with the preservation of liver function [27].

In conclusion, the GNRI‐dNLR score demonstrated robust predictive value for both PFS and OS in unresectable HCC patients treated with Atez/Bev. High GNRI‐dNLR scores were associated with significantly shorter PFS and OS compared to low scores, underscoring the combined prognostic impact of nutritional and inflammatory status. The dNLR, as a surrogate marker for NLR, was effective in databases lacking lymphocyte count data, broadening its applicability.

## Author Contributions

**Atsushi Naganuma:** conceptualization (equal). **Satoru Kakizaki:** data curation (equal), writing – original draft (equal), writing – review and editing (equal). **Atsushi Hiraoka:** data curation (equal). **Toshifumi Tada:** data curation (equal). **Takeshi Hatanaka:** conceptualization (equal), data curation (equal). **Kazuya Kariyama:** data curation (equal). **Joji Tani:** data curation (equal). **Masanori Atsukawa:** data curation (equal). **Koichi Takaguchi:** data curation (equal). **Ei Itobayashi:** data curation (equal). **Shinya Fukunishi:** data curation (equal). **Kunihiko Tsuji:** data curation (equal). **Toru Ishikawa:** data curation (equal). **Kazuto Tajiri:** data curation (equal). **Hidenori Toyoda:** data curation (equal). **Chikara Ogawa:** data curation (equal). **Hiroki Nishikawa:** data curation (equal). **Takashi Nishimura:** data curation (equal). **Kazuhito Kawata:** data curation (equal). **Hisashi Kosaka:** data curation (equal). **Masashi Hirooka:** data curation (equal). **Yutaka Yata:** data curation (equal). **Hideko Ohama:** data curation (equal). **Hidekatsu Kuroda:** data curation (equal). **Tomomitsu Matono:** data curation (equal). **Tomoko Aoki:** data curation (equal). **Yuki Kanayama:** data curation (equal). **Kazunari Tanaka:** data curation (equal). **Fujimasa Tada:** data curation (equal). **Kazuhiro Nouso:** data curation (equal). **Asahiro Morishita:** data curation (equal). **Akemi Tsutsui:** data curation (equal). **Takuya Nagano:** data curation (equal). **Norio Itokawa:** data curation (equal). **Tomomi Okubo:** data curation (equal). **Taeang Arai:** data curation (equal). **Michitaka Imai:** data curation (equal). **Shinichiro Nakamura:** data curation (equal). **Hirayuki Enomoto:** data curation (equal). **Masaki Kaibori:** data curation (equal). **Yoichi Hiasa:** data curation (equal), supervision (equal). **Masatoshi Kudo:** data curation (equal), supervision (equal). **Takashi Kumada:** conceptualization (equal), supervision (equal).

## Ethics Statement

This retrospective study was approved by the Institutional Ethics Committee of Japanese Red Cross Himeji Hospital (IRB No. 2022‐40) in accordance with the Declaration of Helsinki. Written informed consent was obtained from all patients before treatment and this study received ethical approval for use of an opt‐out methodology.

## Conflicts of Interest

Satoru Kakizaki received research grants from AbbVie. Takeshi Hatanaka received lecture fees from Eisai. Atsushi Hiraoka received lecture fees from Eli Lilly, AstraZeneca, and Chugai. Toshifumi Tada received lecture fees from AbbVie, Eisai, and Chugai. Hidenori Toyoda received lecture fees from Eisai, Chugai, Takeda, Terumo, AbbVie, Gilead, Fujifilm WAKO, and Abbott. Hidekatsu Kuroda received lecture fee from Eisai. Kazuhiro Nouso received honoraria from AbbVie, Aska Pharmaceutical, AstraZeneca, Bayer, Century Medical, Chugai, Covidien, Eisai, Gilead, Kowa, Lilly, and Otsuka; and received research funding from CureApp, Denka, Fuji film, and Medtronic. Masatoshi Kudo received honoraria from Bayer, Chugai, Eisai, Eli Lilly, MSD, and Takeda; and received research funding from AbbVie, EA Pharma, Eisai, GE Healthcare, Gilead Sciences, Otsuka, Sumitomo Dainippon Pharma, Taiho, and Takeda. Toshifumi Tada and Masanori Atsukawa are editorial board members of Hepatology Research. The other authors declare no conflicts of interest associated with this study.

## Data Availability

The data associated with present study are available from the corresponding author upon reasonable request.
